# Identification of SNP Markers in the Glutamate Dehydrogenase (GDH) and Aspartate Aminotransferase 2 (AST2) Associated with Ammonia Nitrogen Tolerance in *Penaeus monodon*

**DOI:** 10.3390/biology14111532

**Published:** 2025-10-31

**Authors:** Yangyang Ding, Jinsong Chen, Song Jiang, Qibin Yang, Yundong Li, Jianhua Huang, Lishi Yang, Jianzhi Shi, Yebing Yu, Shigui Jiang, Falin Zhou

**Affiliations:** 1 Key Laboratory of South China Sea Fishery Resources Exploitation and Utilization, Ministry of Agriculture, South China Sea Fisheries Research Institute, Chinese Academy of Fishery Sciences, Guangzhou 510300, China; dingyangyang93@163.com (Y.D.); chenjs25@mail.sysu.edu.cn (J.C.); tojiangsong@163.com (S.J.); yangqibin1208@163.com (Q.Y.); liyd2019@163.com (Y.L.); huangjianhua@scsfri.ac.cn (J.H.); yangls2016@163.com (L.Y.); shijianzhi1989@163.com (J.S.); jiangsg@21cn.com (S.J.); 2 State Key Laboratory of Biocontrol, Southern Marine Science and Engineering Guangdong Laboratory (Zhuhai), School of Life Sciences, Sun Yat-sen University, Guangzhou 510275, China; 3 Yancheng Institute of Technology, College of Marine and Bioengineering, Yancheng 224051, China; yuyebing2005@126.com

**Keywords:** ammonia nitrogen stress, black tiger shrimp, breeding, genetic mutation, molecular marker

## Abstract

**Simple Summary:**

Black tiger shrimp is a major farmed shrimp species in the world, which is negatively impacted by high ammonia nitrogen levels in high-density aquaculture. This study investigated the genetic basis of ammonia tolerance, focusing on two conserved genes (glutamate dehydrogenase and aspartate aminotransferase 2) involved in ammonia metabolism. Single nucleotide polymorphisms (SNPs) in these two genes were identified, genotyped and analyzed in different shrimp populations. Two specific SNPs (*PmGDH*-1227 and *PmAST2*-132) showed a significant distribution difference between ammonia-susceptible and ammonia-resistant shrimp. This study intuitively represented the interaction of *PmGDH* and *PmAST2* to ammonia nitrogen and developed SNP markers linked to ammonia tolerance in these two genes from black tiger shrimp, providing valuable tools for molecular marker-assisted breeding programs to develop new strains with ammonia tolerance, thereby improving the survival rate and yield of black tiger shrimp under high-density farming conditions.

**Abstract:**

Black tiger shrimp (*Penaeus monodon*) is the largest species of penaeid, being commercially cultured globally, ranking as the second most farmed species in the shrimp industry. However, with the transformation of shrimp aquaculture from semi-intensive to high-density farming, the concentration of ammonia nitrogen in the aquatic environment has increased, severely affecting the growth and survival of shrimps. To increase production efficiency, breeding new strains of shrimp with the trait of tolerance to high ammonia nitrogen is desired in the black tiger shrimp aquaculture. Previous studies have shown that glutamate dehydrogenase (GDH) and aspartate aminotransferase 2 (AST2) play important roles in the metabolism of ammonia nitrogen in crustaceans. In the present study, we conducted synteny analysis of *PmGDH* and *PmAST2* in different species. The interactions of *PmGDH* with ammonium (NH_4_^+^) and *PmAST2* with aspartate were analyzed by docking. To develop molecule markers associated with ammonia nitrogen tolerance, SNPs of *PmGDH* and *PmAST2* were identified by direct sequencing, genotyped by the SNaPshot technique, and characterized through genotype-phenotype association analysis by PLINK software version 1.9 in the three geographical populations, two families with different ammonia tolerance, and 120 susceptible and resistant individuals of black tiger shrimp. The results indicate that the *GDH* and *AST2* genes are evolutionarily conserved in vertebrates, except for the black tiger shrimp, which suggests divergence in selective pressure between invertebrates and vertebrates. Notably, *PmGDH* may interact with NH_4_^+^ via the residue Asp178 within loop 1 in the GdhA domain through a hydrogen bonding interaction, and *PmAST2* may interact with aspartate via helix 1, sheet 1, loop 1, and loop 2, through both hydrogen bonding interactions and a salt bridge interaction. A total of 12 SNPs were detected in the exons of *PmGDH* and *PmAST2*. Among these candidate SNPs, the distributions of *PmGDH*-1227 and *PmAST2*-132 showed both significant differences in the genotype and allele association analysis between susceptible and resistant groups. Haplotype association analysis showed that three haplotypes exhibited significantly different distributions between susceptible and resistant groups. In conclusion, *PmGDH*-1227 and *PmAST2*-132 were associated with ammonia nitrogen tolerance, and these SNP markers are expected to contribute to marker-assisted selection (MAS) breeding programs to obtain new strains of *Penaeus monodon*.

## 1. Introduction

Shrimp farming is an important part of China’s aquaculture industry [1]. However, due to the development of intensive high-density aquaculture and the influence of climate change, the aquatic environment of aquaculture has deteriorated seriously in recent years, causing frequent diseases in aquaculture species. Ammonia nitrogen is the major pollutant in the aquatic environment and is one of the most important water quality parameters to monitor [2]. In an isolated aquaculture system, ammonia is one of the main concerns as it may accumulate over time, primarily from the decomposition of organics and from the excretion of the rearing animals [3]. High concentrations of ammonia may result in the retardation of shrimp growth and can cause tissue damage in gills, skin, and blood circulation; in extreme cases, it can cause shrimp mortality [3,4,5,6]. To increase production efficiency, shrimp with the trait of tolerance to high ammonia nitrogen are desired in shrimp farming. Thus, breeding new strains with this desirable trait has become the key to the sustainable development of the shrimp breeding industry.

With the development of molecular biology, marker-assisted selection (MAS) has breathed new life into the aquaculture breeding industry, for it enables selection to depend on genotypes and improves breeding efficiency [7,8]. Molecular markers can be used to detect quantitative trait loci (QTL) that control complex traits of interest and to select individuals for MAS programs [9]. As the third generation of genetic markers, single-nucleotide polymorphism (SNP) has the advantages of high genetic stability, high abundance and widespread distribution, representativeness, co-dominant, and easy to detect [10]. In recent years, SNPs have been widely used in the MAS breeding programs of aquatic animals. Many SNP markers were found to be associated with a variety of economic traits, such as growth, disease resistance, and ammonia tolerance traits [11,12,13,14,15,16,17]. In *Epinephelus coioides*, 25 SNPs were identified to be correlated with the ammonia tolerance, in which more than half of the SNPs were clustered on chromosomes 9 and 16, and the candidate gene *sgk3* may be critical for its function to activate many ion channels and transporters [18]. In *Litopenaeus vannamei*, a SNP marker C/T_545_ in the hemocyanin gene was identified to be associated with both high growth and ammonia tolerance traits [19]. In *Sinonovacula constricta*, 56 associated SNPs embedded in or adjacent to 236 genes were identified to be associated with ammonia tolerance, in which Na^+^/K^+^-ATPase (NKA) was demonstrated to be involved in NH_4_^+^ excretion to reduce ammonia accumulation [20]. However, given that the ammonia tolerance is a quantitative trait with low heritability, currently developed SNP markers are insufficient for MAS breeding programs, especially in shrimps.

In black tiger shrimp (*Penaeus monodon*), ammonia stress is known to induce severe oxidative damage and trigger apoptosis, as revealed by transcriptome analyses [21]. The underlying mechanisms, further elucidated by single-cell sequencing, involve complex responses in oxidative stress, detoxification metabolism, and immune defense [22]. Specific genes, such as chitinase 4 and 5, have also been implicated in the response to ammonia stress [23,24]. Importantly, ammonia is commonly considered a toxic by-product of amino acid metabolism [25]. Our previous study has demonstrated that glutamate dehydrogenase (GDH) and aspartate aminotransferase 2 (AST2) in *P. monodon* contribute to ammonia detoxification [26,27,28]. Recent studies have proved that ammonia can be recycled through reductive amination catalyzed by GDH to central ammonia acid metabolism [25,29]. GDH is an NAD(P)H-dependent enzyme that can catalyze reductive amination of α-ketoglutarate to glutamate. In this process, excessive ammonia was assimilated, and the efficiency of nitrogen utilization was maximized [25]. Likewise, as a mitochondrial transaminase, AST2, also well-known as glutamate oxaloacetate transaminase 2 (GOT2), can catalyze the reversible reaction between glutamate and oxaloacetate, generating α-ketoglutarate and aspartate (Asp) [30,31,32]. Asp is further utilized to synthesize the amino acid asparagine (Asn) and is incorporated into the urea cycle [33]. This cycle will detoxify cellular NH_4_^+^ through urea excretion. In addition, two SNPs in the coding domain region of the *GDH* gene were significantly associated with ammonia tolerance in *Sinonovacula constricta* [34]. However, the SNP markers in the GDH and AST2 from shrimps have not yet been identified.

Black tiger shrimp is the largest species of penaeid being commercially cultured in many countries, especially in Southeast Asia. Up to now, several SNPs associated with growth, low salt tolerance, and diversity traits have been identified in black tiger shrimp [35,36,37]. However, little is known about the SNP markers associated with ammonia tolerance in the black tiger shrimp. In the present study, an acute ammonia stress test was conducted to test the tolerance capacity of the 28 families of black tiger shrimp to ammonia. The synteny of the *PmGDH* and *PmAST2* in different species and the interactions of *PmGDH* with ammonium and *PmAST2* with Asp were analyzed. The SNPs in the *PmGDH* and *PmAST2* were screened by direct sequencing. SNaPshot was applied to process SNP genotyping in the three geographical populations, the 120 susceptible and resistant individuals, and two families with different ammonia tolerance of the black tiger shrimp. The Hardy–Weinberg equilibrium, the Mendelian genetic ratio, and the genotype-phenotype association analysis of the identified SNPs in the *PmGDH* and *PmAST2* were analyzed. This study focused on the genetic basis of the ammonia tolerance trait and may contribute to MAS breeding programs for black tiger shrimp, aiming to reduce mortality and increase production.

## 2. Materials and Methods

### 2.1. Experimental Animals

The wild black tiger shrimp from different geographic populations were collected from four regions of the world (Figure S1). The Sanya population was collected in Sanya, Hainan province, China; the African population was collected in southern Africa near Mozambique; the Indonesian population was collected in Indonesia; and the Thai population was collected in Phuket, Thailand.

A total of 28 different families of healthy black tiger shrimp were selected and bred by our research group in the experimental base of the South China Sea Fisheries Research Institute (Shenzhen, China). According to our previous study, the ammonia-nitrogen tolerance of the No. 9 family was significantly stronger than that of the No. 3 family; thus, they were selected for SNP genotyping. The ID of the female parent of No. 3 family is ♀61109, which comes from the Thai population, and its male parent, which is numbered ♂61537, also belongs to the Thai population. The ID of the female parent of No. 9 family is ♀61116, which comes from the Thai population, and its male parent, which is numbered ♂61625, belongs to the African population.

### 2.2. Ammonia Nitrogen Stress Test

An ammonia nitrogen stress test was conducted at the Shenzhen base of the South China Sea Fisheries Research Institute. Shrimp (body weight: 8.50 ± 3.01 g, body length: 8.16 ± 1.67 cm) from 28 families of *P. monodon* marked with different fluorescent dyes were used (Figure S2). The shrimp were reared in a cement pond (1.5 m × 1.8 m × 1.5 m) for 14 days before the ammonia nitrogen stress test. Continuous aeration was provided in the rearing ponds, and shrimp were fed with a commercial shrimp diet at 1% body weight per day. Ammonium chloride (NH_4_Cl, analytically pure) was added to control the concentration of ammonia nitrogen, and the 96 h median lethal concentration (LC50) in the ammonia nitrogen stress test was 66 mg/L, which was calculated by preliminary experiments according to previous studies [3,38]. The experiments contained two groups (ammonia nitrogen test group and the control group), each group contained two replicates. In the test group, 2800 shrimp from 28 families were evenly assigned into two cement ponds containing 3 tons of seawater with NH_4_Cl added. In the control group, another 2800 shrimp from 28 families were evenly assigned into two cement ponds containing 3 tons of seawater without NH_4_Cl. During the experimental period, the dissolved oxygen was 6.5 ± 0.03 mg/L, the water temperature was 30 ± 0.5 °C, the pH was 7.0 ± 0.5, and the salinity was 30 ‰. Water in the experimental ponds was replaced every 24 h. The mortality of test shrimp in each pond was recorded, and the dead shrimp were collected every 2 h. The first 60 dead shrimp were defined as the susceptible group, and the last 60 surviving shrimp were defined as the resistant group. Shrimp were stored in 95 % ethanol. Shrimp were anesthetized on ice before sampling. All efforts were made to minimize the suffering of the shrimp.

### 2.3. Syntenic Analysis

JCVI was performed to explore gene synteny within 5 species at the whole genome level, and the results were visualized using TBtools 2.0. The genome annotation files of *P. monodon* (GCF_015228065.2), *Homo sapiens* (GCF_000001405.40), *Mus musculus* (GCA_000001635.9), *Xenopus tropicalis* (GCF_000004195.4) and *Danio rerio* (GCA_000002035.4) were downloaded from the NCBI database and inter-species protein sequence alignment was conducted using the same method to detect sequence homology, with the E-value < 1 × 10^−5^ and the minimum number of gene pairs set to 10. The gene synteny maps composed of *GDH*, *AST2*, and its neighbor genes of other species were learned by the browser of Genomicus v110.01 (https://www.genomicus.bio.ens.psl.eu/genomicus-110.01/cgi-bin/search.pl, accessed on 25 August 2025).

### 2.4. Protein-Ligand Docking

The *PmGDH* (XP_037799881.1) and *PmAST2* (XP_037778756.1) protein sequence was obtained from the NCBI database, and their structures were modeled using AlphaFold3 software. The energy of the proteins was optimized using Rosetta Relax, and their structural preparations, such as dehydrogenation and hydrogenation, were performed on the acquired 3D structure of the proteins. The structures of NH_4_^+^ and aspartic acid (Asp) were obtained from the PubChem database (https://pubchem.ncbi.nlm.nih.gov/). The *PmGDH*-NH_4_^+^ complex and the *PmAST2*-Asp complex were modeled using blind docking on the CB-DOCK2 online server (https://cadd.labshare.cn/cb-dock2/php/blinddock.php, accessed on 4 July 2025), and docking was made using Autodock Vina 1.2.3. The protein-ligand force analysis was conducted by relying on the PLIP online server (https://plip-tool.biotec.tu-dresden.de/plip-web, accessed on 4 July 2025) analysis, and the protein-ligand 3D model was displayed using Pymol software version 2.5.4.

### 2.5. Sampling and DNA Extraction

Muscular tissues were dissected separately from fresh shrimp and stored immediately in 95% ethanol until the DNA samples were isolated. The total DNA was extracted from all collected tissues using the HiPure Tissue DNA Mini Kit (Magen, D3121, Guangzhou, China) according to the manuals. The concentration of DNA was measured by a NanoDrop 2000 spectrophotometer (Thermo Fisher Scientific, Shanghai, China). The integrity of DNA was assessed by electrophoresis on 1% agarose gel. The DNA samples were diluted to 100 ng/μL and stored at −20 °C.

### 2.6. Screen of SNPs in the PmGDH and PmAST2

The genome sequences of *PmGDH* (LOC119594874) and *PmAST2* (LOC119575290) were obtained in the NCBI database, and the predicted genome structures were illustrated by IBS 2.0 software. According to their genome sequences, the potential domain region of *PmGDH* and *PmAST2* was selected for further polymorphism analysis. Then Sanger sequencing was used to screen SNPs on exon 5 and exon 6 of *PmGDH*, and exon 1 and exon 2 of *PmAST2*. A total of 32 DNA samples were randomly selected from four geographic populations (Sanya, Indonesian, African, and Thai populations) of P. monodon as templates, and specific primers (Table S1) were designed to amplify specific sequence fragments mentioned above. PCR products were purified and sent to Beijing Genomics Institute (BGI, Shenzhen, China) for sequencing. Vector NTI Advance^®^ 11.5 software was used to align the sequencing results to obtain SNPs.

### 2.7. Genotyping of SNPs in the PmGDH and PmAST2

After screening and evaluation, all the available SNPs obtained via the methods above were genotyped in 376 shrimp, which contained 50 individuals from African population, 56 individuals from Indonesian population, 50 individuals from Thai population, 50 individuals from the No. 3 family, 50 individuals from the No. 9 family, 60 individuals from the susceptible group and 60 individuals from the resistant group, using Multiplex SNaPshot system (Ruibiotech, Beijing, China) based on ABI PRISM 3730 XI Genetic Analyzer platform (Applied Biosystems, Woburn, MA, USA). The primers for SNPs amplification and extension primers for Multiplex SNaPshot genotyping were listed in Table S2. The SPSS 23.0 software (IBM, Armonk, NY, USA) was used to analyze the Mendelian genetic ratio in No. 3 and No. 9 families. Popgene 32 Version 1.32 software was used to verify the Hardy–Weinberg equilibrium of the three geographic groups.

### 2.8. Association Analysis Between Candidate SNPs and Ammonia Nitrogen Tolerance

To search for the SNP markers associated with ammonia nitrogen tolerance, the candidate SNPs were genotyped in the susceptible group and the resistant group by SNaPshot genotyping mentioned above. Only the SNP loci with the minor allele frequency (MAF) greater than 1% would be applied to the association analysis [39]. Differences in the distributions of genotype frequency of each SNP locus in susceptible and resistant groups were assessed using Pearson’s chi-square test, which was performed with statistical software SPSS 23.0. Distributions of allele frequency of each SNP locus in susceptible and resistant groups were analyzed by PLINK software version 1.9 with Fisher models. The odds ratios (OR) of alleles were also calculated. Linkage disequilibrium and haplotype-trait association analysis were analyzed using the Haploview software package v4.2 [40]. The test for normality and homogeneity of variances was performed before analysis.

### 2.9. Statistical Analysis

The survival rate in the ammonia nitrogen stress test was analyzed using the one-way ANOVA method followed by Tukey’s multiple comparison test in SPSS 23.0 and presented as mean ± the standard error of the mean (mean ± SEM). Figures were made using GraphPad Prism 8 software. It was considered statistically significant when *p* < 0.05 and highly statistically significant when *p* < 0.01.

## 3. Results

### 3.1. Susceptible and Resistant Families in the Ammonia-Nitrogen Stress Test

**Figure 1:**
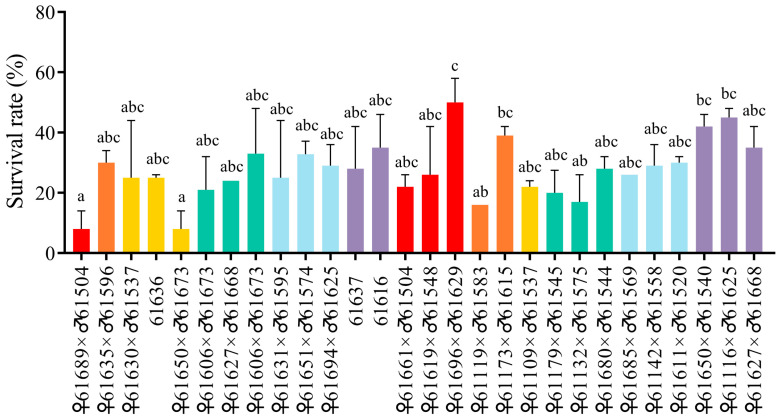
The 48 h survival rate of 28 families in *P. monodon* in the ammonia nitrogen test. The histogram represents the 48 h survival rate (%) of 28 families in *P. monodon* after ammonia nitrogen stress. The horizontal axis represents the number of parents in the families. Values (expressed as mean ± SEM, *n* = 100) with different letters are significantly different from each other (*p* < 0.05).

The ammonia nitrogen stress test lasted for about 128 h until all the shrimp from 28 families were dead. The first dead shrimp was observed approximately 2 h after ammonia nitrogen stress, and the mortality was increasing gradually until peaking at 24 h. The survival rate of 28 families at 48 h was calculated and presented in Figure 1. The most resistant family is the ♀61696 × ♂61629 group, whose female parent and male parent both belong to the African strain. The most susceptible family is the ♀61689 × ♂61504 group, whose female parent is from the African strain and male parent is from the Thailand strain, and the ♀61650 × ♂61673 group, whose female parent and male parent both belong to the African strain. The 48 h survival rate of the ♀61696 × ♂61629 group, the ♀61689 × ♂61504 group, and the ♀61650 × ♂61673 group is 50%, 8% and 8%, respectively. Accordingly, 60 shrimp that died within the first 8 h were classified into the susceptible group for their high sensitivity to ammonia nitrogen, and the last 60 shrimp that survived over 104 h post challenge were considered as resistant individuals. All the shrimp were still alive during the whole period in the control group.

### 3.2. Synteny Analysis of PmGDH and PmAST2

**Figure 2:**
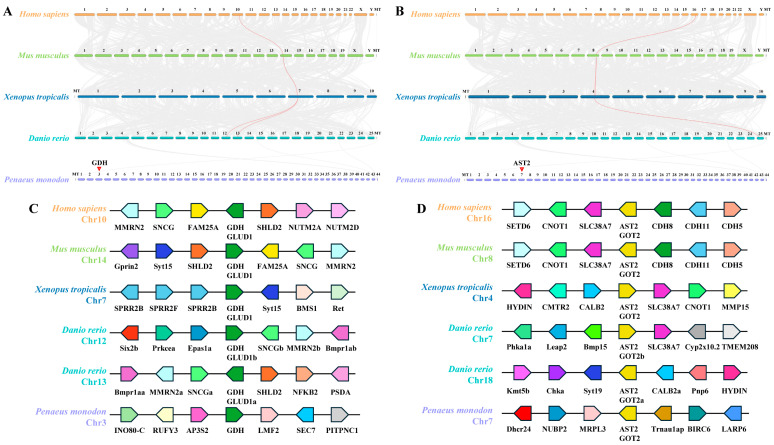
Syteney analysis of *PmGDH* and *PmAST2*. (**A**) Synteny analysis across the genomes of *P. monodon*, *Homo sapiens*, *Mus musculus*, *Xenopus tropicalis*, and *Danio rerio*. Syntenic blocks between *P. monodon* and other species’ genomes were represented by gray lines, while red lines highlight the syntenic relationship of the *GDH* gene. (**B**) Synteny analysis across the genomes of *P. monodon*, *Homo sapiens*, *Mus musculus*, *Xenopus tropicalis*, and *Danio rerio*. Syntenic blocks between *P. monodon* and other species’ genomes were represented by gray lines, while red lines highlight the syntenic relationship of the *AST2* gene. (**C**) Analysis of the *GDH* gene collinearity in representative species. The same genes were represented with the same color, and the direction of transcription was indicated by the acute angle of the pentagon. (**D**) Analysis of *AST2* gene collinearity in representative species. The same genes were represented with the same color, and the direction of transcription was indicated by the acute angle of the pentagon.

In *P. monodon*, the GDH gene is located on chromosome 3, and the AST2 gene is located on chromosome 7. Synteny analysis between *P. monodon* and other species (*Homo sapiens*, *Mus musculus*, *Xenopus tropicalis*, and *Danio rerio*) revealed 1, 1, 1, and 2 GDH genes with synteny. However, the GDH synteny relationship between *Danio rerio* and *P. monodon* did not exist (Figure 2A). Similarly, the synteny analysis between *P. monodon* and other species revealed 1 AST2 gene. But the synteny relationship of *AST2* between *Danio rerio* and *P. monodon* did not exist (Figure 2B). The lack of synteny of GDH and AST2 in *P. monodon* and other species may be due to the divergence of selective pressure in invertebrates and vertebrates. In addition, gene synteny analysis exhibited that genes neighboring the GDH gene and AST2 are at variance. Mostly, adjoining genes of the GDH gene in *Homo sapiens*, *Mus musculus*, and *Danio rerio* are SNCG and MMRN2, respectively (Figure 2C). In addition, adjoining genes of the AST2 gene in *Homo sapiens*, *Mus musculus*, and *Xenopus tropicalis* are SLC38A7 and CNOT1, respectively, and SLC38A7 is also conserved in *Danio rerio* (Figure 2D). Notably, there are 2 GDH genes (GLUD1a on Chr13 and GLUD1b on Chr12) and 2 AST2 genes (GOT2a on Chr18 and GOT2b on Chr7) in the *Danio rerio* (Figure 2C,D).

### 3.3. The PmGDH-NH_4_^+^ Complex and the PmAST2-Asp Complex

**Figure 3:**
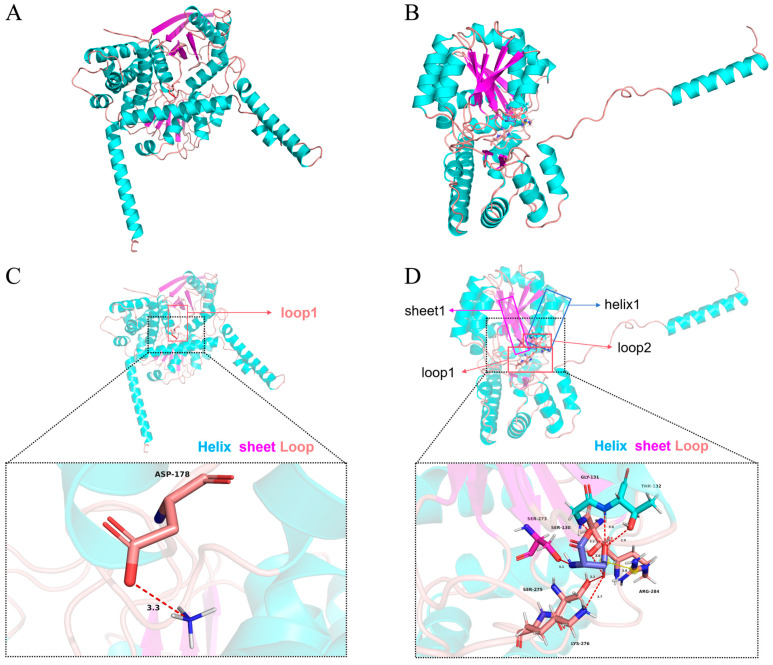
The predicted structural model of *PmGDH*-NH_4_^+^ complex and *PmAST2*-Asp complex. (**A**) Predicted structural model of the *PmGDH*-NH_4_^+^ complex. The secondary structures of the *PmGDH* protein are shown in different colors. Helix, blue. Sheet, red. Loop, orange. (**B**) Predicted structural model of the *PmAST2*-Asp complex. The secondary structures of the *PmGDH* protein are shown in different colors. Helix, blue. Sheet, red. Loop, orange. (**C**) A detailed view of the binding mode of the *PmGDH* protein to the ammonium ion (NH_4_^+^). The interacting residue Asp178 on loop 1 of *PmGDH* and NH_4_^+^ are colored, respectively. The pink atoms are O atoms of Asp178 of *PmGDH*, and the dark blue atoms are N atoms of NH_4_^+^. The hydrogen bonding interaction is indicated in a red dotted line. The number next to the dotted line indicates the force bond length, measured in angstroms (Å). (**D**) A detailed view of the binding mode of the *PmAST2* protein to the aspartic acid (Asp) molecule. The interacting residues of *PmAST2* and Asp are colored, respectively. The red atoms are O atoms, the dark blue atoms are N atoms, and the light blue atoms are C atoms of protein residues. The purple atoms represent the C atoms of the Asp molecules. The hydrogen bonding interactions are indicated in red dotted lines, and the salt bridge interaction is indicated in a yellow dotted line. The numbers next to the dotted lines indicate the force bond length, measured in angstroms (Å). Three replicates were performed to predict the complex structure models.

To intuitively demonstrate the interaction of *PmGDH* and *PmAST2* with ammonia nitrogen, molecular docking was performed. To identify the key amino acids involved in the binding of *PmGDH* to ammonium ion and *PmAST2* to aspartic acid, the interactions in the *PmGDH*-NH_4_^+^ complex and the *PmAST2*-Asp complex were analyzed. Upon protein-ligand docking, a hydrogen bonding interaction was formed between the *PmGDH* protein and NH_4_^+^ (Figure 3A,C). Within loop 1 of the *PmGDH* protein, amino acid residue Asp178 forms a hydrogen bonding interaction with NH_4_^+^ at a distance of 3.3 Å (Figure 3C). The binding energy between the *PmGDH* protein and NH_4_^+^ was predicted to be −1.4 kcal/mol according to Vina’s binding energy prediction algorithm based on the AMBER force field. Thus, we hypothesized that loop 1 in the GdhA domain of *PmGDH* might be involved in its binding to NH_4_^+^. Meanwhile, the binding interface between the *PmAST2* protein and aspartic acid encompasses predominantly hydrogen bonding interactions, with the addition of a salt bridge interaction (Figure 3B,D). Aspartic acid can interact with helix 1, sheet 1, loop 1, and loop 2 of the *PmAST2* protein. Aspartic acid can form hydrogen bonding interactions with amino acid residues Thr132 and Gly131 of helix 1, Ser273 of sheet 1, Ser130 of loop 1, Ser275 and Lys276 of loop 2 in the *PmAST2* protein at 2.9/3.0 Å, 3.2 Å, 3.1 Å, 3.0 Å, 3.2 Å, and 3.7 Å, respectively (Figure 3D). Aspartic acid can also form a salt-bridge interaction with amino acid residue Arg284 of loop 2 in the *PmAST2* protein at 3.8 Å (Figure 3D). The binding energy between the *PmAST2* protein and aspartic acid was predicted to be −5.6 kcal/mol according to Vina’s binding energy prediction algorithm based on the AMBER force field. Thus, we hypothesized that helix 1, sheet 1, loop 1, and loop 2 in the AAT_I (Aspartate aminotransferase superfamily fold type I) domain of *PmAST2* might be involved in its binding to aspartic acid.

### 3.4. Identification of SNPs in Exons of PmGDH and PmAST2

**Figure 4:**
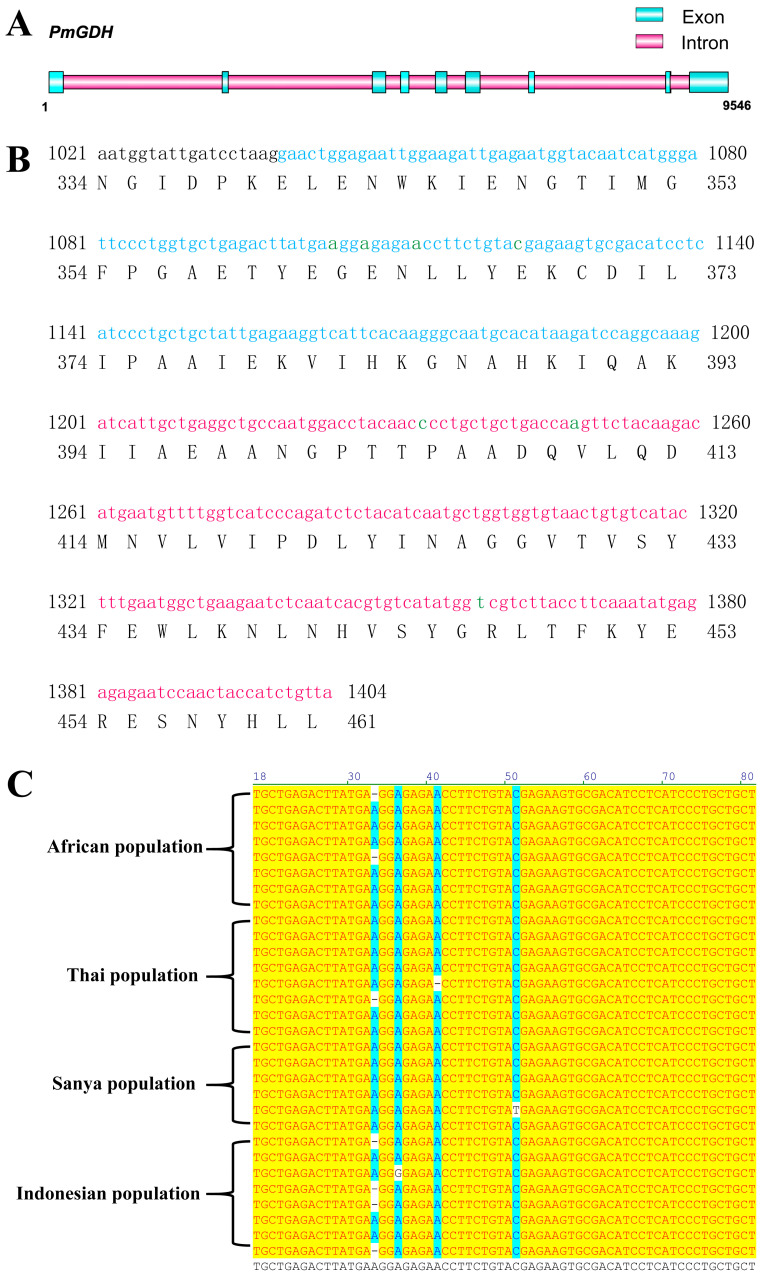
SNPs in exon 5 and exon 6 of *PmGDH*. (**A**) Genome structure of *PmGDH*, including 9 exons (blue) and 8 introns (red). (**B**) Nucleotide and amino acid sequences of exon 5 and exon 6 of *PmGDH*. Exon 5 is marked in blue. Exon 6 is marked in red. SNPs are marked in green. The serial number on both sides of each row refers to the location of the nucleotides and amino acids. (**C**) Alignment of the nucleotide from exon 5 of *PmGDH* in the different individuals belonging to the African group, Thai group, Sanya group, and Indonesian group, respectively. SNPs in exon 5 are marked in blue.

**Figure 5:**
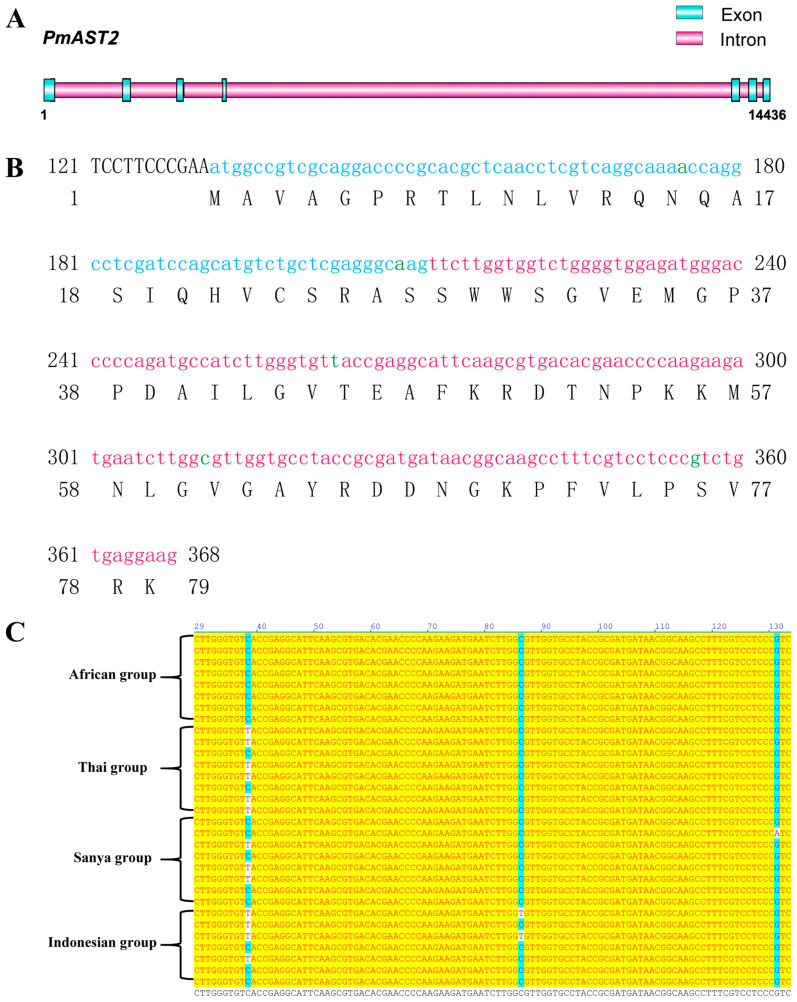
SNPs in exon 1 and exon 2 of *PmAST2*. (**A**) Genome structure of *PmAST2*, including 7 exons (blue) and 6 introns (red). (**B**) Nucleotide and amino acid sequences of exon 1 and exon 2 of *PmAST2*. Exon 1 is marked in blue. Exon 2 is marked in red. SNPs are marked in green. The serial number on both sides of each row refers to the location of the nucleotides and amino acids. (**C**) Alignment of the nucleotide from exon 2 of *PmAST2* in the different individuals belonging to the African group, Thai group, Sanya group, and Indonesian group, respectively. SNPs in exon 2 are marked in blue.

The complete genome sequence of the *PmGDH* gene was approximately 9546 bp, including 9 exons and 8 introns (Figure 4A). The results of direct sequencing showed that a total of seven SNPs were detected in *PmGDH*, including four SNPs in exon 5 and three SNPs in exon 6 (Figure 4B). The base count was noted as 1 at the beginning of the initiation codon. Among them, *PmGDH*-1086, *PmGDH*-1101, and *PmGDH*-1227 loci were transitions, *PmGDH*-1212 and *PmGDH*-1338 were transversions, and *PmGDH*-1083 and *PmGDH*-1091 loci were deletion loci (Table 1). Alignment of the nucleotide from exon 5 of *PmGDH*, including *PmGDH*-1083, *PmGDH*-1086, *PmGDH*-1091, and *PmGDH*-1101, in the different individuals belonging to the African group, Thai group, Sanya group, and Indonesian group was shown in Figure 4C. The complete genome sequence of *PmAST2* is about 14,436 bp, including 7 exons and 6 introns (Figure 5A). There are two SNPs in exon 1 and three SNPs in exon 2 of *PmAST2*, respectively *(*Figure 5B), including deletion loci *PmAST2*-44, transversion loci *PmAST2*-78, and transition loci *PmAST2*-132, *PmAST2*-180, and *PmAST2*-225 (Table 1). Alignment of the nucleotide from exon 2 of *PmAST2*, including *PmAST2*-132, *PmAST2*-180, and *PmAST2*-225, in the different individuals belonging to the African group, Thai group, Sanya group, and Indonesian group was shown in Figure 5C. The minor allele frequency (MAF) of all SNP loci is greater than 1% in this study, and most of them are synonymous mutations.

### 3.5. Genotyping of Potential SNPs

According to the standard of longer flanking sequence, better quality, and suitable for designing SNaPshot primer, *PmGDH*-1101, *PmGDH*-1212, *PmGDH*-1227, *PmAST2*-132, and *PmAST2*-225 loci were chosen for SNaPshot genotyping. The No. 3 and No. 9 families were applied to evaluate the Mendelian inheritance of five polymorphic SNPs. The results of SNP genotyping in families are shown in Table 2. In the No. 9 family, parental genotypes of all the loci were homozygous except *PmAST2*-132 with parental genotype CC × CT, among which *PmGDH*-1101 and *PmAST2*-132 were not in line with Mendel’s laws of inheritance (*p* < 0.05). In No. 3 family, the parental genotypes of *PmGDH*-1101, *PmGDH*-1212, and *PmAST2*-132 were heterozygous, and the parental genotypes of *PmGDH*-1227 and *PmAST2*-225 were homozygous. In the No. 3 family, all SNP loci were segregated and assorted independently, fitting well with Mendel’s inheritance law.

The African, Indonesian, and Thai geographic populations were used to verify the Hardy–Weinberg equilibrium of each SNP locus (Table 3). In the African population, almost all SNP loci were deviated significantly from the Hardy–Weinberg equilibrium at *p* < 0.01 except *PmGDH*-1227. Alleles of all the SNP loci are spread relatively evenly in the Indonesian population, and all of them are in accordance with the Hardy–Weinberg equilibrium. In the Thai population, genotyping results of all SNP loci showed significant fitness to the Hardy–Weinberg equilibrium except *PmAST2*-132 (*p* < 0.05).

### 3.6. SNPs Are Associated with Ammonia Nitrogen Tolerance in P. monodon

The results of the association analysis with the chi-square test and Fisher model are shown in Table 4 and Table 5. Two SNPs were identified to be associated with ammonia nitrogen tolerance. In detail, the genotype frequency distributions of *PmGDH*-1101, *PmGDH*-1227, *PmAST2*-132, and *PmAST2*-225 were significantly different between the susceptible group and the resistant group (*p* < 0.01). And the allele frequency distributions of *PmGDH*-1227 and *PmAST2*-132 were also significantly different between the susceptible group and the resistant group (*p* < 0.05, FDR < 0.05, OR < 1). Shrimp containing these alleles would be more resistant to ammonia nitrogen. These two SNPs in *PmGDH* and *PmAST2*, associated with ammonia nitrogen tolerance, were further analyzed to investigate the Linkage disequilibrium of the SNPs. The results showed that the SNPs located on the same gene fragment were in Linkage disequilibrium.

Haplotype association analysis showed that there were three haplotypes whose frequency distributions were significantly different between resistant and susceptible groups (*p* < 0.01) (Table 6). The haplotype frequencies of *PmGDH*-1227-G/*PmAST2*-132-C in the resistant group were significantly higher than those in the susceptible group (*p* < 0.01), while the frequencies of *PmGDH*-1227-A/*PmAST2*-132-T and *PmGDH*-1227-A/*PmAST2*-132-C in the resistant group were significantly lower than those in the susceptible group (*p* < 0.05).

## 4. Discussion

High concentrations of ammonia nitrogen in water can inhibit the excretion of ammonia nitrogen in shrimps, leading to an increased concentration of ammonia nitrogen in their hemolymph. This condition results in a decrease in hemocyanin content and an increase in free amino acid levels, ultimately causing hypoxia, metabolic disorders, and reduced immunity in shrimps [41,42]. Given that different individuals and families of shrimp may exhibit varying tolerances to ammonia, family selection is a practical traditional breeding method in which the entire family is considered as a selection unit. This approach is particularly effective for phenotypic traits with low heritability, such as fecundity, survival rates, and resistance [3]. In this study, we compared the survival rate of 28 black tiger shrimp families to evaluate their tolerance capacity to ammonia nitrogen stress. The most resistant family, whose female parent and male parent are numbered ♀61,696 × ♂61,629, was obtained. This family can be utilized in future family selection for new strains of black tiger shrimp with ammonia nitrogen resistance, and the shrimp aquaculture industry to maximize commercial productivity. The susceptible and resistant individuals were also obtained, which is useful for the genotyping and genotype-phenotype association analysis of SNP markers associated with ammonia tolerance.

Glutamate dehydrogenase (GDH) can catalyze the oxidative deamination of glutamate, and aspartate aminotransferase 2 (AST2) is an efficient catalyst for ammonia transfer reaction; they all play important roles in the metabolism of ammonia nitrogen in crustaceans [43,44]. The results of synteny analysis revealed that the GDH and AST2 genes are evolutionarily conserved in vertebrates, suggesting the same function of GDH and AST2 in the detoxification of ammonia in vertebrates. However, synteny between black tiger shrimp and vertebrates was not observed, including GDH and AST2, suggesting that the invertebrates and vertebrates might cope with different selective pressures. The ligand-protein docking revealed the interaction models between *PmGDH* and ammonium and between *PmAST2* and amino acids, elucidating the mechanisms by which GDH and AST2 exert their ammonia detoxification functions. In mammals, as a ubiquitous by-product of cellular metabolism, excess ammonia can be recycled directly by GDH to build new amino acids, such as glutamate, which also plays an important role in ammonia detoxification by forming glutamine [25]. In addition, the deficiency of GOT2 increased ammonia levels in mice [45].

In the present study, a total of 12 SNPs in exons of *PmGDH* and *PmAST2* were identified by direct sequencing; most of them were synonymous mutations. Previous studies generally agree that in the genome, especially in the coding region of genes, synonymous mutations are far more common than non-synonymous mutations. This is due to the biological evolution mechanism that tends to have the self-elimination of harmful mutations [46]. The *PmGDH* and *PmAST2* genes are relatively conserved and strongly influenced by negative selection, so the frequency of synonymous mutations in the encoding region is higher than that of non-synonymous mutations [26,27,47]. Then, five eligible SNPs were chosen to be genotyped in 376 shrimp by the SNaPshot genotyping technique, and the success rate of genotyping is 94.9%. SNaPshot is a highly accurate genotyping method that allows for the simultaneous detection of multiple sites and can identify contaminated samples, making it suitable for projects with medium throughput and large sample sizes [48]. According to the result of genotyping in families and geographical populations, several SNPs in the No. 9 family and the African population were deviated significantly from Mendel’s inheritance law and Hardy–Weinberg equilibrium. In this study, the No. 9 family is stronger in ammonia nitrogen tolerance than the No. 3 family. In addition, the African population is genetically distant from the Southeast Asian populations [37,49]. In this situation, SNPs in these groups are influenced more frequently by environmental selection, and the allele frequencies of SNPs associated with ammonia nitrogen tolerance are higher than normal.

In the previous studies of other species, the loci associated with QTLs always showed allelic heterogeneity between the analyzed groups with significantly different phenotypes [50,51,52]. Similar results were shown for some ammonia nitrogen tolerance-associated makers in this research. After marker-trait association analysis using Pearson’s chi-square test, Fisher model, and Logistic model, three SNP markers were identified to be associated with ammonia nitrogen tolerance. The genotype frequency distributions of four SNPs were significantly different in the resistant group compared to the susceptible group (*p* < 0.05). The allele frequency distributions of three SNP loci showed a significant difference between susceptible and resistant groups (*p* < 0.05). Among these candidate SNPs, the distributions of *PmGDH*-1227 and *PmAST2*-132 showed both significant differences with the genotype and allele analysis between the susceptible and resistant groups (*p* < 0.05). In *Litopenaeus vannamei*, 12 SNP markers from the 1826 ammonia-responsive genes were identified to be associated with ammonia tolerance, containing 10 loci with significantly different allele frequencies and 10 loci with significantly different genotyping frequencies [53]. Another study in *Litopenaeus vannamei* has identified six SNPs to be significantly associated with ammonia tolerance, and 7 candidate genes (*pdi*, *ozf*, *upf2*, *vps16*, *tmem19*, *mycbp2*, and *hox7*) were retrieved around these SNPs [54]. Haplotype association analysis showed that three haplotypes exhibited significantly different distributions between susceptible and resistant groups (*p* < 0.05). It is of note that alleles of different SNPs associated with ammonia nitrogen tolerance tend to appear in one individual simultaneously. Shrimp with these SNPs may be stronger in tolerance of ammonia nitrogen, and these two SNPs may be appropriate breeding markers for new strains of black tiger shrimp.

This study has many limitations, such as the sample sizes for SNP screens and association analysis. In future studies, we will employ larger numbers of extreme individuals for genotype-phenotype association analyses, such as 200–300 individuals per group. We will also adopt more advanced methods like genome-wide association study (GWAS) to analyze ammonia nitrogen tolerance loci at the genome-wide level, alongside more rigorous quality control and data analysis methodologies. The loci *PmGDH*-1227 and *PmAST2*-132 found in this study will also be validated further for applying them to MAS programs.

## 5. Conclusions

In conclusion, 12 SNP markers were identified in the exons of *PmGDH* and *PmAST2* in this study, in which *PmGDH*-1227 and *PmAST2*-132 proved to be associated with ammonia nitrogen tolerance of black tiger shrimp. To improve the accuracy of results, SNP genotyping was performed using the SNaPshot method in two families, three different geographic populations, and the susceptible and resistant individuals. The genotype-phenotype association analysis was processed by PLINK with Pearson’s chi-square test, logistic regression, and Fisher models. This is the first report for the SNP markers associated with ammonia nitrogen tolerance in *P. monodon*. These SNP markers provided important potential for MAS and will be applied to construct genetic linkage maps and breed new strains of black tiger shrimp with ammonia nitrogen tolerance.

## Figures and Tables

**Table 1:** Characteristics of the 12 polymorphic SNP loci enrolled in this study.

SNP ID	Location	Position from Start Codon (bp)	Mutation Type	Variation	Amino Acid
*PmGDH*-1083	Exon 5 of *PmGDH*	1083	Absence	GGA	Glycine
*PmGDH*-1086	Exon 5 of *PmGDH*	1086	Transition, synonymous	GGA/GGG	Glycine
*PmGDH*-1091	Exon 5 of *PmGDH*	1091	Absence	AAC	Asparagine
*PmGDH*-1101	Exon 5 of *PmGDH*	1101	Transition, synonymous	TAC/TAT	Tyrosine
*PmGDH*-1212	Exon 6 of *PmGDH*	1212	Transversion, synonymous	ACC/ACA	Threonine
*PmGDH*-1227	Exon 6 of *PmGDH*	1227	Transition, synonymous	CAA/CAG	Glutamine
*PmGDH*-1338	Exon 6 of *PmGDH*	1338	Transversion, synonymous	GGT/GGA	Glycine
*PmAST2*-44	Exon 1 of *PmAST2*	44	Absence	AAC	Asparagine
*PmAST2*-78	Exon 1 of *PmAST2*	78	Transversion, synonymous	GCA/GCT	Alanine
*PmAST2*-132	Exon 2 of *PmAST2*	132	Transition, synonymous	GTT/GTC	Valine
*PmAST2*-180	Exon 2 of *PmAST2*	180	Transition, synonymous	GGC/GGT	Glycine
*PmAST2*-225	Exon 2 of *PmAST2*	225	Transition, synonymous	CCG/CCA	Proline

**Table 2:** SNP genotyping of *PmGDH* and *PmAST2* in No. 3 and No. 9 families.

Family ID	Sample Size	SNP ID	Parents (♀ × ♂)	Genotype Frequency (%)		*p*-Value for Mendelian Ratio	Chi-Square Value
No. 9	50	*PmGDH*-1101	TT × CC	CT 96.0	CC 4.0	0.043 *	4.082
	50	*PmGDH*-1212	CC × CC	CC 100	\	\	\
	50	*PmGDH*-1227	GG × GG	GG 100	\	\	\
	50	*PmAST2*-132	CC × TC	CT 96.0	TT 4.0	0.000 **	64.438
	50	*PmAST2*-225	GG × GG	GG 100	\	\	\
No. 3	49	*PmGDH*-1101	CT × CC	CC 44.9	CT 55.1	0.479	0.501
	49	*PmGDH*-1212	CA × CC	CA 49.0	CC 51.0	0.888	0.020
	49	*PmGDH*-1227	GG × GG	GG 98.0	GA 2.0	0.155	2.020
	49	*PmAST2*-132	TT × CT	TT 40.8	CT 59.2	0.201	1.633
	49	*PmAST2*-225	GG × GG	GG 98.0	GA 2.0	0.155	2.020

Significant differences are indicated with an asterisk: * *p* < 0.05, ** *p* < 0.01.

**Table 3:** SNP genotyping of *PmGDH* and *PmAST2* in three geographic groups.

Geographic Groups	Sample Size	SNP ID	Genotype Frequency (%)			*p*-Value for Hardy–Weinberg Equilibrium	Chi-Square Value
African group	50	*PmGDH*-1101	TT 10.0	CT 6.0	CC 84.0	<0.01 **	29.20
	50	*PmGDH*-1212	AA 2.0	AC 2.0	CC 96.0	<0.01 **	32.33
	50	*PmGDH*-1227	GA 8.0	GG 92.0		0.79	0.06
	50	*PmAST2*-132	TT 6.0	CT76.0	CC 18.0	<0.01 **	22.17
	50	*PmAST2*-225	AA 4.0	GA 8.0	GG 88.0	<0.01 **	14.95
Indonesian group	56	*PmGDH*-1101	TT 33.9	CT 41.1	CC 25.0	0.18	1.83
	56	*PmGDH*-1212	AC 10.7	CC 89.3		0.70	0.15
	56	*PmGDH*-1227	AA 1.8	GA 35.7	GG 62.5	0.35	0.86
	56	*PmAST2*-132	TT 44.6	CT 51.8	CC 3.6	0.08	3.17
	56	*PmAST2*-225	AA 1.8	GA 28.6	GG 69.6	0.70	0.15
Thai group	50	*PmGDH*-1101	TT 38.0	CT 42.0	CC 20.0	0.31	1.01
	50	*PmGDH*-1212	AA 2.0	AC 20.0	CC 78.0	0.65	0.21
	50	*PmGDH*-1227	GA 0.32	GG 0.68		0.19	1.69
	50	*PmAST2*-132	TT 42.0	CT 56.0	CC 2.0	0.02 *	5.27
	50	*PmAST2*-225	GA 28.0	GG 72.0		0.27	1.22

Significant differences are indicated with an asterisk: * *p* < 0.05, ** *p* < 0.01.

**Table 4:** Genotype frequency of SNPs in *PmGDH* and *PmAST2* in the association analysis with ammonia nitrogen tolerance.

SNP ID	Genotype	Genotype Frequency (%)		Chi-Square Value	*p*-Value
		RG	SG		
*PmGDH*1101	CT	12 (20.3)	6 (8.6)	4.88	0.027 *
	CC	47 (79.7)	52 (91.4)		
*PmGDH*1212	AC	2 (3.4)	5 (8.6)	3.191	0.074
	CC	57 (96.6)	53 (91.4)		
*PmGDH*1227	GA	8 (13.8)	20 (34.5)	28.151	0.000 **
	GG	48 (82.8)	28 (48.3)		
	AA	2 (3.4)	10 (17.2)		
PmAST132	TC	11 (18.3)	16 (28.1)	12.908	0.002 **
	CC	40 (66.7)	24 (42.1)		
	TT	9 (15.0)	17 (29.8)		
*PmAST2*25	GA	2 (3.3)	8 (14.0)	9.517	0.009 **
	GG	57 (95.0)	46 (80.7)		
	AA	1 (1.7)	3 (5.3)		

RG: resistant group; SG: susceptible group; Significant differences are indicated with an asterisk: * *p* < 0.05, ** *p* < 0.01.

**Table 5:** Allele frequency of SNPs in *PmGDH* and *PmAST2* in the association analysis with ammonia nitrogen tolerance.

SNP ID	A1	F_A	F_U	A2	MAF	NCHROBS	Frequency	*p*-Value for Fisher	OR	FDR
*PmGDH*-1101	T	0.1017	0.0431	C	0.0727	234	0.9274	0.1290	2.5130	0.3606
*PmGDH*-1212	A	0.0170	0.0431	C	0.0299	234	0.9701	0.2785	0.3828	0.3606
*PmGDH*-1227	A	0.1034	0.3448	G	0.2241	232	0.7759	0.0000 **	0.2192	0.0001 **
*PmAST2*-132	T	0.2417	0.4386	C	0.3376	234	0.6624	0.0015 **	0.4079	0.0077 **
*PmAST2*-225	A	0.0333	0.1228	G	0.0769	234	0.9231	0.0131 *	0.2463	0.0522

A1: Minor allele name; F_A: Frequency of A1 in resistant group; F_U: Frequency of A1 in susceptible group; A2: Major allele name; MAF: Minor allele frequency; NCHROBS: Number of alleles at the observed SNP; OR: Odds ratio; Significant differences are indicated with an asterisk: * *p* < 0.05, ** *p* < 0.01.

**Table 6:** Haplotype analysis of SNPs involved in ammonia nitrogen tolerance in resistant and susceptible groups.

Locus	Haplotype	Susceptible Frequency (%)	Resistant Frequency (%)	Chi-Square Value	*p*-Value
*PmGDH*-1227/ *PmAST2*-132	GC	99 (45.0)	163 (70.3)	29.567	0.000 **
	GT	43 (19.5)	45 (19.4)	0.002	0.968
	AT	49 (22.3)	11 (4.7)	26.511	0.000 **
	AC	29 (13.2)	13 (5.6)	7.694	0.006 **

** *p* < 0.01, significantly different distributions of the haplotype frequency between these two groups.

## Data Availability

The original data can be downloaded at Zenodo, https://doi.org/10.5281/zenodo.17454008.

## Supplementary Materials

The following supporting information can be downloaded at: https://www.mdpi.com/article/10.3390/biology14111532/s1, Figure S1: Map of sampling sites of geographical groups of *P. monodon*; Figure S2: Shrimp from different families of *P. monodon* marked with fluorescent dyes; Table S1: Primers used in detecting SNPs on *PmGDH* and *PmAST2*; Table S2: Primers used for Multiplex SNaPshot genotyping.
