# Supplementary material for: Identification of SNP Markers in the Glutamate Dehydrogenase (GDH) and Aspartate Aminotransferase 2 (AST2) Associated with Ammonia Nitrogen Tolerance in Penaeus monodon
Source: Biology (Basel). 2025 Oct 31;14(11):1532. doi: 10.3390/biology14111532 (PMC12650449; doi:10.3390/biology14111532)
Supplement: Supplementary file 1 [file biology-14-01532-s001.zip › Supplementary material.pdf]

Figure

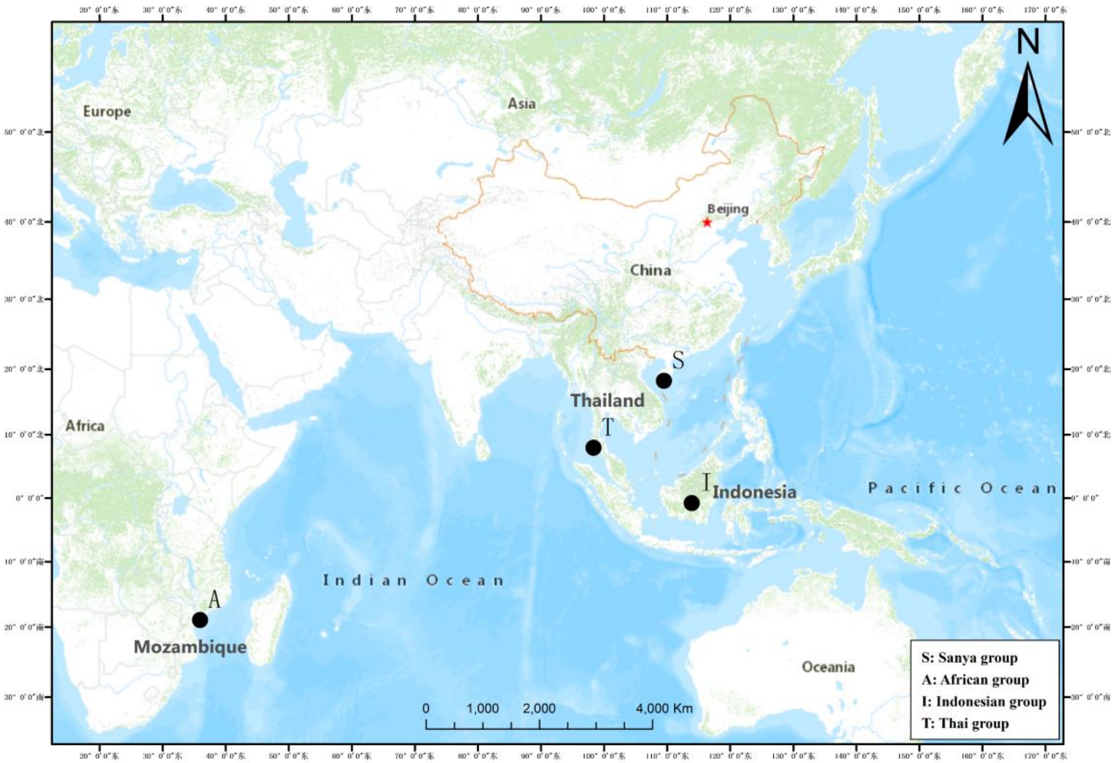

Figure S1. Map of sampling sites of geographical groups of *Penaeus monodon*.

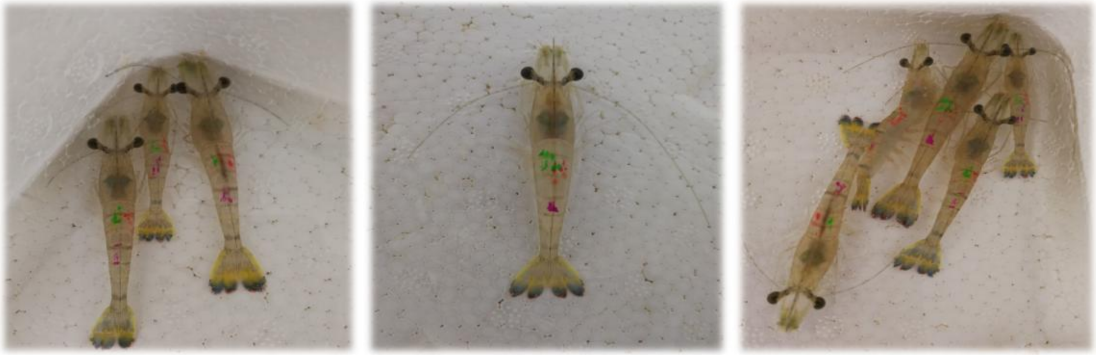

Figure S2. Shrimp from different families of *Penaeus monodon* marked with fluorescent dyes.

Table

Table S1

Primers used in detecting SNPs on *PmGDH* and *PmAST2*.

Primer	Sequence (5’-3’)	Amplification target
PmAST2-E1F	AAATACCTTTCGTCCTTC	Exon 1 of *PmAST2*
PmAST2-E1R	TACACTTAGTCTTGACAAT	
PmAST2-E2F	ACAGGTAGGATTAGTAAG	Exon 2 of *PmAST2*
PmAST2-E2R	TCTCTATTTCTAAGCATTG	
PmGDH-E5F	GGTAATAACTATTGTAACAG	Exon 5 of *PmGDH*
PmGDH-E5R	CAACCATTAAGACACTAA	
PmGDH-E6F	TTGCTAGTTGATTGGTAA	Exon 6 of *PmGDH*
PmGDH-E6R	GTAGATGTGTAATTGTGTTA	

Table S2

Primers used for Multiplex SNaPshot genotyping.

Primer	Sequence (5’-3’)	Function
1-F	ACTTTTTCCAGGAACTGGAG	Amplification of exon 5
1-R	ACCTAACAGATGGTAGTTGG	and 6 in *PmGDH*
1-573-r	TTTTTTTTTTTTTTTTTTTTTTTTTATGAGGATGTCGCACTTCTC	Detection of PmGDH1101
1-944-f	TTTTTTTTTTTTTTTTTTTTTTTTTTTTTTGCTGCCAATGGACCTACAAC	Detection of PmGDH1212
1-958-r	TTTTTTTTTTTTTTTTTTTTTTTTTTTTTTTTTTACATTCATGTCTTGTAGAAC	Detection of PmGDH1227
2-2-F	GCCGAGAGAACTAAACTAATAACTT	Amplification of exon 2 in
2-2-R	AGAGAAAAAACAATATATAAATGAAAGAGG	*PmAST2*
2-1055-R	TTTTTTTTTTTTTTTTTTTTTCACGCTTGAATGCCTCGGT	Detection of PmAST132
2-1149-f	TTTTTTTTTTTTTTTTTTTTTTTTTTTTTTTTTTTTTTTTGGCAAGCCTTTCGTCCTCCC	Detection of PmAST225
